# Integrated nontargeted and targeted metabolomics analyses amino acids metabolism in infantile hemangioma

**DOI:** 10.3389/fonc.2023.1132344

**Published:** 2023-03-21

**Authors:** Kaiying Yang, Tong Qiu, Xue Gong, Jiangyuan Zhou, Yuru Lan, Siyuan Chen, Yi Ji

**Affiliations:** ^1^ Division of Oncology, Department of Pediatric Surgery, West China Hospital of Sichuan University, Chengdu, Sichuan, China; ^2^ Department of Pediatric Surgery, Guangzhou Women and Children’s Medical Center, National Children’s Medical Center for South Central Region, Guangzhou Medical University, Guangzhou, China; ^3^ Pediatric Intensive Care Unit, Department of Critical Care Medicine, West China Hospital of Sichuan University, Chengdu, China

**Keywords:** infantile hemangioma, hemangioma-derived endothelial cell, amino acids, nontargeted metabolic, targeted metabolic

## Abstract

Infantile hemangioma (IH) is the most common benign tumor in children. However, the exact pathogenesis of IH remains unclear. Integrated nontargeted and targeted metabolic analyses were performed to obtain insight into the possible pathogenic mechanism of IH. The results of nontargeted metabolic analysis showed that 216 and 128 differential metabolites (DMs) were identified between hemangioma-derived endothelial cells (HemECs) and HUVECs in positive-ion and negative-ion models, respectively. In both models, these DMs were predominantly enriched in pathways related to amino acid metabolism, including aminoacyl-tRNA biosynthesis and arginine and proline metabolism. Then, targeted metabolic analysis of amino acids was further performed to further clarify HemEC metabolism. A total of 22 amino acid metabolites were identified, among which only 16 metabolites, including glutamine, arginine and asparagine, were significantly differentially expressed between HemECs and HUVECs. These significant amino acids were significantly enriched in 10 metabolic pathways, including ‘alanine, aspartate and glutamate metabolism’, ‘arginine biosynthesis’, ‘arginine and proline metabolism’, and ‘glycine, serine and threonine metabolism’. The results of our study revealed that amino acid metabolism is involved in IH. Key differential amino acid metabolites, including glutamine, asparagine and arginine, may play an important role in regulating HemEC metabolism.

## 1 Introduction

Infantile hemangioma (IH) is the most common benign tumor in children, with a prevalence of 4%-5% (1). The growth pattern that characterizes IH involves a proliferating phase for 1 year after birth, followed by spontaneous involution for several years. Most IHs are small and self-resolving; therefore, treatment is not necessary (2). However, approximately 10% of IHs are potentially problematic and can cause significant complications and morbidities, including ulceration, obstruction, functional impairment and disfigurement (3). Although β-adrenergic receptor antagonists, including propranolol, atenolol, and timolol, have exhibited remarkable efficacy in IH treatments, the exact pathogenesis of IH is not well understood (3–6).

In the proliferating phase of IH, hemangioma-derived endothelial cells (HemECs) are the predominant cell type. It is widely accepted that endothelial cell (EC) angiogenesis plays a vital role in the development of IH (7). ECs are stimulated by factors such as vascular endothelial growth factor (VEGF) and hypoxia; thus, ECs have the capability to rapidly switch their metabolic state from a quiescent state to an angiogenic state, resulting in the activation of EC angiogenesis (8). To date, EC metabolism has been recognized as a driving force of angiogenesis (9). Several metabolic pathways are involved in EC metabolism, including glucose metabolism (glycolysis), fatty acid oxidation, and amino acid metabolism. Our previous study revealed that glucose metabolism may play an important role in the pathogenesis of IH (10). However, little is known about the metabolic profiles and role of HemEC in the development of IH.

Metabolomics aims to measure a wide variety of small molecules (metabolome) in the context of physiological stimuli or disease states (11). With the development of mass spectrometry, metabolomics has become a valuable approach to identify biomarkers for diagnosis, prognosis, or treatment efficacy (12). Thus, the present study was performed to investigate the metabolic profiles of HemECs and to understand the complex mechanism underlying the development of IH using integrated nontargeted and targeted metabolomic analysis. Our results suggest that amino acid metabolism is involved in HemEC metabolism.

## 2 Material and methods

### 2.1 Cell culture and reagents

Endothelial basal medium (EBM-2, CC-3156) containing the EGM-2 growth factors (CC-4176) was obtained from Lonza (Walkersville, MD, USA). EC medium (ECM) was obtained from ScienCell (CA, USA). Fetal bovine serum (FBS), phosphate-buffered saline (PBS), penicillin and streptomycin were purchased from Gibco (Grand Island, NY, USA).

HemEC isolation was performed as described previously (13, 14). Totally, tissues from six proliferating IHs and four involuting IHs were collected. Detailed information on the six patients is presented in **Table S1**. The HemECs were cultured in EBM-2 containing 10% FBS, 100 U/ml penicillin and 10 µg/ml streptomycin at 37°C containing 5% CO_2_. Human umbilical vein ECs (HUVECs) were obtained from the Chinese Academy of Sciences and cultured in ECM (ScienceCell) plus 10% FBS and with EGM-2 growth factors (CC-4176). This study was approved by the Ethics Committee of the West China Hospital of Sichuan University. The design of our research in this text was approved by the West China Hospital of Sichuan University.

### 2.2 Nontargeted metabolomics analysis using ultrahigh-performance liquid chromatography

#### 2.2.1 Sample collection and preparation

HemECs and HUVECs were cultured in EBM-2 and ECM medium, respectively. Then, after 24 hours in culture, the cells were collected and stored at -80°C before further processing for UHPLC analysis.

#### 2.2.2 Metabolite extraction

After the samples were freeze-dried, two hundred microliters of water was added. After 30 s of vortexing, the samples were thawed with liquid nitrogen 3 times. The samples were sonicated for 10 min in an ice water bath. Fifty microliters of homogenate was used to measure the protein concentration. Different volume samples were transferred to an EP tube, and water was replenished to 150 μL. See the attached table for details. Then, 600 μL acetonitrile: methanol (1: 1) with an isotopically labeled internal standard mixture was added and transferred to a 2 mL EP tube. After 30 s of vortexing, the samples were incubated at -40°C for 1 h and centrifuged at 12000 (RCF=13800 g, R=8.6 cm) rpm for 15 min at 4°C. Then, 700 μL of supernatant was transferred to an EP tube and dried in a vacuum concentrator. Next, 80 μL acetonitrile: methanol: water = 2: 2: 1 was added to the sample. After 30 s of vortexing, the samples were sonicated for 10 min in an ice-water bath. Then, the samples were centrifuged at 12000 (RCF=1380 (×g), R= 8.6 cm) rpm for 15 min at 4 °C. The resulting supernatant was transferred to a fresh glass vial for analysis. A quality control (QC) sample was prepared by mixing an equal aliquot of the supernatants from all samples.

#### 2.2.3 LC−MS/MS analysis

LC−MS/MS analyses were performed using an UHPLC system (Vanquish, Thermo Fisher Scientific) with a UPLC BEH Amide column (2.1 mm × 100 mm, 1.7 μm) coupled to a Q Exactive HFX mass spectrometer (Orbitrap MS, Thermo). The mobile phase consisted of 25 mmol/L ammonium acetate and 25 ammonia hydroxide in water (pH = 9.75) (A) and acetonitrile (B). The autosampler temperature was 4°C, and the injection volume was 4 μL.

The QE HFX mass spectrometer was used for its ability to acquire MS/MS spectra in information-dependent acquisition (IDA) mode under the control of acquisition software (Xcalibur, Thermo). In this mode, the acquisition software continuously evaluates the full scan MS spectrum. The ESI source conditions were set as follows: sheath gas flow rate, 30 Arb; Aux gas flow rate, 25 Arb; capillary temperature, 350 °C; full MS resolution, 120,000; MS/MS resolution, 7500; collision energy, 10/30/60 in NCE mode; and spray voltage, 3.6 kV (positive) or -3.2 kV (negative).

#### 2.2.4 Data preprocessing and annotation

The raw data were converted to the mzXML format using ProteoWizard and processed with an in-house program, which was developed using R and based on XCMS, for peak detection, extraction, alignment, and integration. Then, an in-house MS2 database (BiotreeDB) was applied for metabolite annotation. The cutoff for annotation was set at 0.3.

### 2.3 Targeted metabolomics analysis of amino acids

#### 2.3.1 Sample collection and preparation

HemECs and HUVECs were cultured in EBM-2 and ECM medium, respectively. Then, after 24 hours in culture, the cells were collected and stored at -80°C before being further processed for UHPLC−MS/MS analysis.

#### 2.3.2 Metabolite extraction

The samples were centrifuged at 1500 rpm (RCF=216(×g), R= 8.6 cm) and 4°C for 5 min to remove PBS. After 300 μL of water was added, the samples were vortexed for 30 s. The samples were precooled in dry ice and subjected to repeated freeze−thaw cycles three times in liquid nitrogen. The samples were vortexed for 30 s and sonicated for 15 min in an ice-water bath. A 250 μL aliquot of clear supernatant was transferred to a new EP tube before a 1000 μL aliquot of extract solvent (1:1 acetonitrile-methanol containing isotopically labeled internal standard mixture, precooled at -40 °C) was added. The remaining sample supernatant was measured for protein content. After the samples were incubated at -40 °C for one hour and centrifuged at 12000 rpm (RCF=13800 (×g), R= 8.6 cm) and 4 °C for 15 min, an 80 μL aliquot of the clear supernatant was transferred to an autosampler vial for UHPLC−MS/MS analysis.

#### 2.3.3 UHPLC-MRM-MS analysis

UHPLC separation was carried out using an Agilent 1290 Infinity II series UHPLC System (Agilent Technologies) equipped with a Waters ACQUITY UPLC BEH Amide column (100 × 2.1 mm, 1.7 μm). Mobile phase A was 1% formic acid in water, and mobile phase B was 1% formic acid in acetonitrile. The column temperature was set at 35°C. The autosampler temperature was set at 4°C, and the injection volume was 1 μL.

An Agilent 6460 triple quadrupole mass spectrometer (Agilent Technologies) equipped with an AJS electrospray ionization (AJS-ESI) interface was applied for assay development. The typical ion source parameters were capillary voltage = +4000/-3500 V, nozzle voltage = +500/-500 V, gas (N_2_) temperature = 300°C, gas (N_2_) flow rate = 5 L/min, sheath gas (N_2_) temperature = 250°C, sheath gas flow rate = 11 L/min, and nebulizer pressure = 45 psi.

The MRM parameters for each of the targeted analytes were optimized using flow injection analysis by injecting the standard solutions of the individual analytes into the API source of the mass spectrometer. Several of the most sensitive transitions were used in MRM scan mode to optimize the collision energy for each Q1/Q3 pair. Among the optimized MRM transitions per analyte, the Q1/Q3 pairs that showed the highest sensitivity and selectivity were selected as ‘quantifiers’ for quantitative monitoring. The additional transitions acted as ‘qualifiers’ to verify the identity of the target analytes.

Agilent MassHunter Work Station Software (B.08.00, Agilent Technologies) was employed for MRM data acquisition and processing.

### 2.4 Statistical analysis

Univariate analysis was conducted, including Student’s t test and fold change (FC) analysis. Multivariate analysis included unsupervised principal component analysis (PCA) and orthogonal partial least squares discriminant analysis (OPLS-DA). The variable importance in the projection (VIP) value (>1) in the OPLS-DA model and P value analyzed by Student’s t test were combined to confirm the significance of differential metabolites (DMs). DMs were considered significant when VIP > 1 and P value < 0.05. Kyoto Encyclopedia of Genes and Genomes (KEGG) pathway enrichment analysis for significant DMs was conducted by the MetaboAnalyst online tool (15, 16).

## 3 Results

### 3.1 Nontargeted metabolomics analysis between HUVECs and HemECs

#### 3.1.1 Multivariate statistical analysis

**Figure 1:**
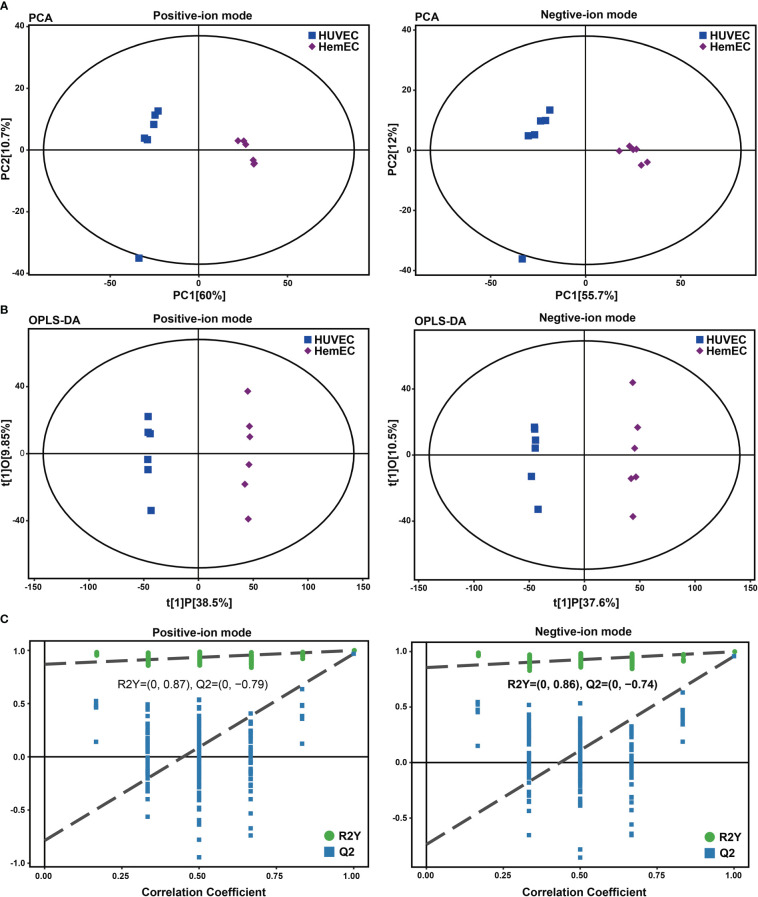
Score plots of principal component analysis (PCA), orthogonal partial least squares discriminant analysis (OPLS-DA) and single-dimensional statistical analysis between the HUVEC group and HemEC group by nontargeted metabolic analysis. **(A)** PCA score plots between HUVECs and HemECs in both positive-ion and negative-ion modes. **(B)** OPLS-DA score plots between HUVECs and HemECs in both modes. **(C)** Permutation tests consisting of 200 permutations demonstrated that the OPLS-DA model was not overfitted in either mode.

To analyze the metabolic changes between HUVECs and HemECs, nontargeted metabolomics analysis was performed using LC−MS/MS. Plots for the unsupervised PCA score showed a clear separation between HUVECs and HemECs in both positive-ion and negative-ion modes (**Figure 1A**). Compared to PCA, supervised OPLS-DA is a more appropriate model to differentiate origins when many factors can affect metabolite profiles (17). Then, OPLS-DA was conducted to further differentiate the metabolic profiles of HUVECs and HemECs. The results showed that HUVECs were also separated clearly from HemECs by the OPLS-DA score plots in both modes (**Figure 1B**). Additionally, further permutation tests demonstrated that the model was not overfitted (positive-ion model: R2Y= (0.0, 0.87), Q2Y= (0.0, -0.79); negative-ion model: R2Y= (0.0, 0.86), Q2Y= (0.0, -0. 74); **Figure 1C**). Together, these data indicated that the metabolic change between HUVECs and HemECs was significant.

#### 3.1.2 Analysis of differential metabolites between the HUVEC group and HemEC group

**Figure 2:**
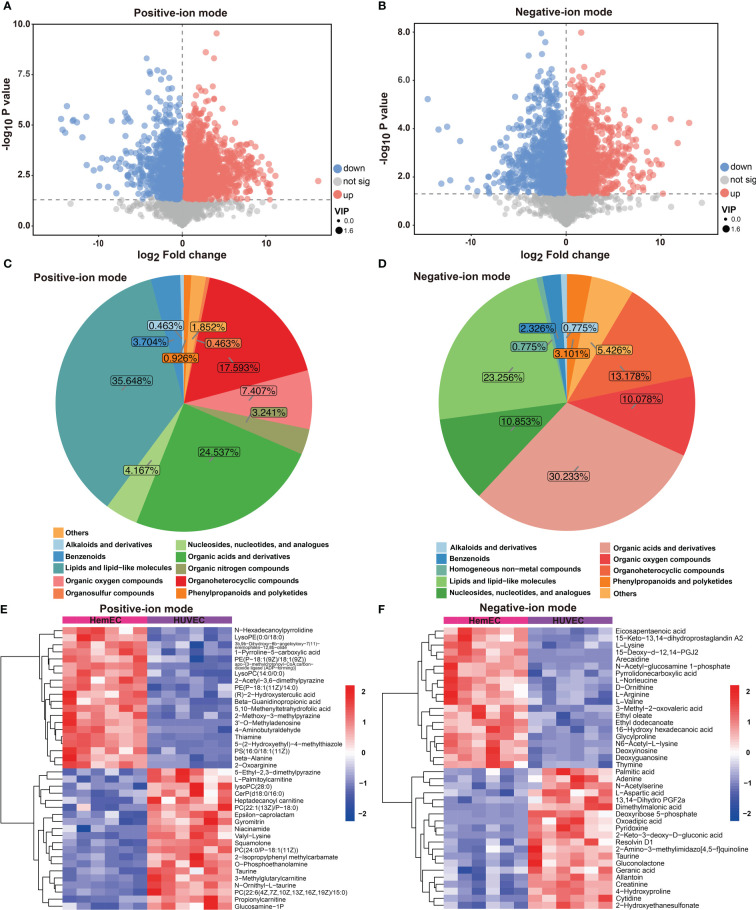
Differential metabolites (DMs) between HUVECs and HemECs by nontargeted metabolic analysis. **(A, B)** Volcano plot analysis of DMs with VIP value > 1 and P value <0.05 in both modes. **(C, D)** Distribution of all DMs in both modes. **(E, F)** Heatmap of hierarchical cluster analysis of the top 30 upregulated and downregulated DMs in both modes.

To compare differences in metabolites between HUVECs and HemECs, differential metabolites (DMs) were screened out in both positive-ion and negative-ion modes. As shown in **Figure 2A**, a total of 216 DMs, including 83 downregulated and 133 upregulated DMs, were identified in positive-ion mode (**Table S2**). These DMs were classified into eleven groups (**Figure 2C**), including lipids and lipid-like molecules (35.648%) and organic acids and derivatives (24.537%). Similarly, 128 DMs were identified between the two groups in negative-ion mode, including 54 downregulated and 74 upregulated DMs (**Figure 2B**, **Table S3**). Most of these DMs were organic acids and derivatives or lipids and lipid-like molecules, which accounted for 30.233% and 23.256% of the DMs, respectively (**Figure 2D**). Then, a heatmap was generated *via* hierarchical clustering analysis for the top 30 DMs, and upregulation and downregulation were observed in both modes (**Figures 2E, F**). The results showed that these DMs were predominantly classified into amino acids and their derivatives, suggesting that amino acid metabolism may be tightly associated with HemEC metabolism. In addition, we used chord plots to visualize the correlation between classifications and the content of metabolites (**Figure S1**). High correlations were observed between different metabolites in the same group of compounds, such as the correlations between beta-alanine and aspartyl-asparagine (**Figure S1A**). In addition, correlations were present between different metabolites from different groups of compounds (**Figures S1A, B**). These results indicated that different metabolites were tightly interconnected. In summary, these data confirmed that there were significant differences in the metabolites between HUVECs and HemECs.

#### 3.1.3 Metabolic enrichment analysis and pathway analysis

**Figure 3:**
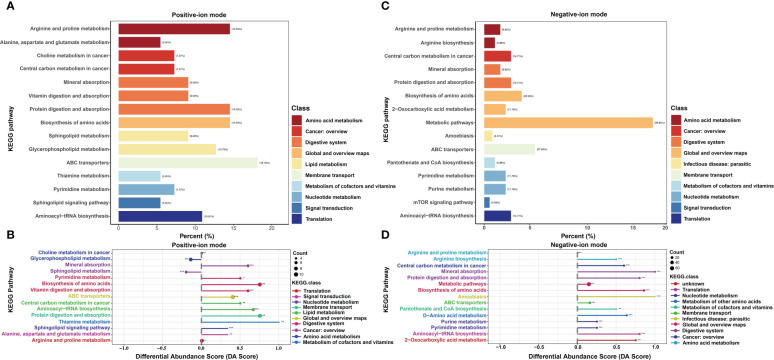
KEGG enrichment analysis of differential metabolites (DMs) between HUVECs and HemECs by nontargeted metabolic analysis. **(A, C)** Enrichment analysis of DMs. **(B, D)**, Differential abundance score analysis of DMs. Each dot represents a metabolic pathway. The X-axis is the differential abundance (DA) score, and the Y-axis is the ID number of the KEGG metabolic pathway. * represents significance. * P < 0.05, ** P < 0.01, *** P < 0.001.

In positive-ion mode, pathway enrichment analysis was performed using the metabolic sets, and the results showed that these DMs were predominantly enriched in ABC transport (18.18%), followed by biosynthesis of amino acids (14.55%), protein digestion and absorption (14.55%) and arginine and proline metabolism (14.55%), as shown in **Figure 3A**. Then, differential abundance score analysis was further performed to determine the overall changes in all DMs enriched in the same pathway. The pathways, including glycerophospholipid metabolism and sphingolipid metabolism, tended to be significantly downregulated. However, the metabolism of various amino acids, including thiamine, pyrimidine, alanine, aspartate and glutamate, tended to be significantly upregulated (**Figure 3B**). Similarly, in the negative-ion model, these DMs were predominantly enriched in metabolic pathways (92.65%), ABC transport (27.94%) and biosynthesis of amino acids (20.59%) (**Figure 3C**). The differential abundance score analysis results showed that all pathways were significantly increased except for arginine and proline metabolism (**Figure 3D**).

**Figure 4:**
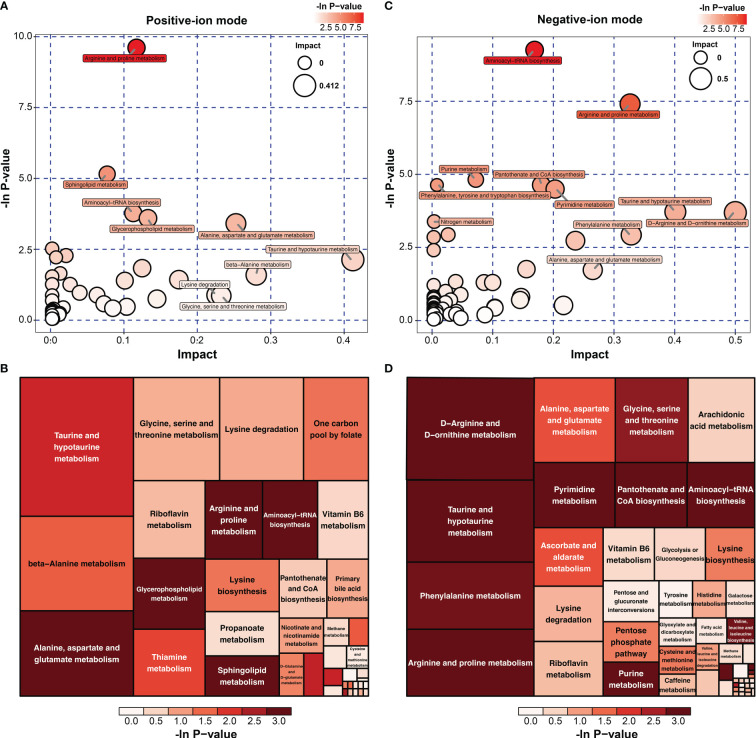
KEGG pathway analysis of differential metabolites (DMs) between HUVECs and HemECs by nontargeted metabolic analysis. **(A, C)** Bubble plot analysis of pathways. Each related metabolic pathway is shown as a circle, in which the size and color are based on the pathway impact value and the P value, respectively. **(B, D)** Treemap plot analysis of pathways. Each related metabolic pathway is shown as a rectangle, in which the size and color are based on the pathway impact value and the P value, respectively.

To investigate the metabolomic pathways involved in IH development, all DMs were enriched. A total of 39 metabolic pathways were identified, as shown in **Figure 4A** (**Table S4**). These metabolism pathways were mainly classified into amino acid metabolism (e.g., arginine and proline metabolism), lipid metabolism (e.g., riboflavin metabolism), fatty acid metabolism and glycerophospholipid metabolism. However, the amino acid metabolism-related pathway was the predominant pathway, including ‘arginine and proline metabolism’, ‘arginine and proline metabolism’, and ‘alanine, aspartate and glutamate metabolism’ (**Figure 4B**). Among the 39 metabolism pathways, only 5 pathways were significant with p < 0.05 and impact >0.1, including ‘arginine and proline metabolism’, ‘Sphingolipid metabolism’, ‘aminoacyl-tRNA biosynthesis’, ‘glycerophospholipid metabolism’ and ‘alanine, aspartate and glutamate metabolism’ (**Table 1**). Similarly, 52 metabolic pathways were identified (**Table S5**), among which 6 pathways were significantly enriched with p < 0.05 and impact >0.1 (**Table 2**). In addition, the amino acid metabolism-related pathway was the predominant pathway in the negative-ion mode (**Figures 4C, D**). Furthermore, two metabolic pathways, ‘aminoacyl-tRNA biosynthesis’ and ‘arginine and proline metabolism’, were significantly enriched in both the positive-ion model and negative-ion model. Notably, these pathways were mainly associated with amino acid metabolism.

**Table 1:** Significant pathways in positive-ion model.

Pathway	Total	Hits	P value	FDR	Impact
Arginine and proline metabolism	77	10	0.00007	0.00537	0.11714
Sphingolipid metabolism	25	4	0.00579	0.23143	0.07726
Aminoacyl-tRNA biosynthesis	75	6	0.02289	0.52974	0.11268
Glycerophospholipid metabolism	39	4	0.02748	0.52974	0.13276
Alanine, aspartate and glutamate metabolism	24	3	0.03311	0.52974	0.25261

**Table 2:** Significant pathways in negative-ion model.

Pathway	Total	Hits	P value	FDR	Impact
Aminoacyl-tRNA biosynthesis	75	10	0.00010	0.00767	0.16902
Arginine and proline metabolism	77	9	0.00061	0.02426	0.32655
Pantothenate and CoA biosynthesis	27	4	0.00971	0.14873	0.18014
Pyrimidine metabolism	60	6	0.01116	0.14873	0.20281
Taurine and hypotaurine metabolism	20	3	0.02425	0.24962	0.40108
D-Arginine and D-ornithine metabolism	8	2	0.02496	0.24962	0.50000

### 3.2 Targeted metabolomics analysis of amino acids

#### 3.2.1 Multivariate statistical analysis

**Figure 5:**
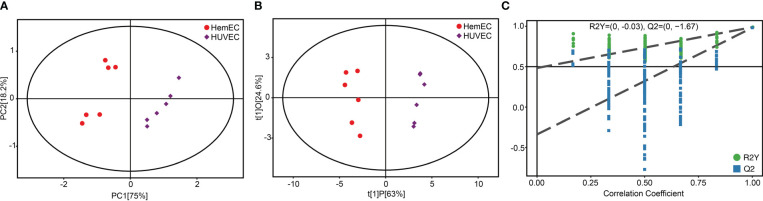
Score plots of principal component analysis **(A)**, orthogonal partial least squares discriminant analysis **(B)** and single-dimensional statistical analysis **(C)** between the HUVEC group and HemEC group by targeted metabolic analysis.

Previous results indicated that the altered metabolic pathways between HUVECs and HemECs were mainly involved in amino acid metabolism pathways. Then, a target amino acid metabolomics analysis was further conducted to gain more insight into HemEC metabolism. Based on the PCA results, a significant separation was observed between the HUVEC group and HemEC group (**Figure 5A**). Additionally, the OPLS-DA showed clearly distinguished profiles between the HUVEC group and HemEC group (**Figure 5B**). Permutation tests consisting of 200 permutations revealed that the model was not overfitted (R^2^Y= (0.0, -0.03), Q^2^= (0.0, -1.67) (**Figure 5C**).These data suggested that altered amino acid profiles could discriminate HemECs from HUVECs.

#### 3.2.2 Differential amino acid metabolites between HUVECs and HemECs

**Figure 6:**
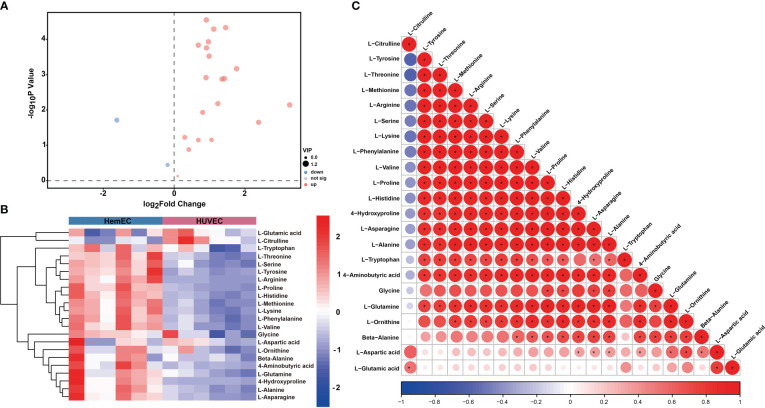
Differential metabolites (DMs) between HUVECs and HemECs by targeted metabolic analysis. **(A)** Volcano plot analysis of DMs with VIP value > 1 and P value <0.05. **(B)** Heatmap of hierarchical cluster analysis of 22 DMs. **(C)** Correlation analysis of 22 differential amino acids. The color of each line represents the Pearson correlation coefficient of two differential metabolites. Red indicates a positive correlation, and blue indicates a negative correlation. *represents significance. P < 0.05.

To compare the differences in metabolites between HUVECs and HemECs, a total of 22 amino acid metabolites were identified (**Table 3**), as presented in the volcano plot (**Figure 6A**). Then, a heatmap was generated *via* hierarchical clustering analysis for all metabolites (**Figure 6B**). In total, 16 amino acids were significantly differentially expressed, including L-glutamine, L-asparagine, L-serine and L-lysine, in the HemEC group compared to the HUVEC group (P < 0.05, **Table 3**). To further clarify the mutual regulatory relationship between metabolites, correlation analysis was performed to reveal the synergy of changes between metabolites (**Figure 6C**). It was obvious that L-glutamine, a vital amino acid in regulating angiogenesis (18), was only negatively correlated with L-citrulline but was significantly positively correlated with the remaining amino acids except for L-tryptophan. Based on these data, significant differences were observed in the metabolites between the propranolol group and the control group.

**Table 3:** Twenty two differential amino acid between HUVEC and HemEC by targeted metabolic analysis.

Metabolite	HemEC	HUVEC	VIP	P value	Q value	FC
L-Methionine	2391.80009	1274.35897	1.18722	0.00003	0.00037	1.87687
L-Histidine	2303.96209	840.50517	1.16469	0.00005	0.00037	2.74116
L-Lysine	10156.39624	4679.73702	1.18294	0.00005	0.00037	2.17029
L-Valine	7627.10054	3903.30832	1.13975	0.00012	0.00064	1.95401
L-Phenylalanine	3528.05398	2200.93489	1.14356	0.00015	0.00064	1.60298
L-Serine	10221.55640	5427.13231	1.15111	0.00017	0.00064	1.88342
L-Threonine	4545.12084	2295.78360	1.15944	0.00030	0.00094	1.97977
L-Proline	17041.78291	5026.96534	1.17854	0.00068	0.00187	3.39007
L-Tyrosine	3064.77825	1643.32832	1.18847	0.00121	0.00267	1.86498
L-Arginine	9453.68867	3535.66444	1.20138	0.00129	0.00267	2.67381
L-Alanine	19159.04603	7510.44802	1.06543	0.00134	0.00267	2.55099
L-Asparagine	7050.21815	2999.44481	1.06503	0.00660	0.01210	2.35051
4-Hydroxyproline	3105.71075	324.16326	1.16101	0.00717	0.01214	9.58070
L-Glutamine	64618.42141	36967.56288	0.90606	0.01168	0.01836	1.74798
L-Citrulline	30.37834	93.19105	0.87037	0.01933	0.02835	0.32598
4-Aminobutyric acid	190.12271	36.45004	1.00520	0.02232	0.03069	5.21598
L-Tryptophan	306.45723	249.04303	0.79563	0.05951	0.07701	1.23054
Beta-Alanine	807.25129	388.58974	0.56343	0.06988	0.08209	2.07739
L-Ornithine	1270.26266	791.34975	0.70438	0.07090	0.08209	1.60518
Glycine	13269.95634	9950.54744	0.72262	0.13132	0.14445	1.33359
L-Glutamic acid	32662.35677	37124.35167	0.37251	0.35882	0.37590	0.87981
L-Aspartic acid	5506.94650	5137.17752	0.00374	0.74428	0.74428	1.07198

#### 3.2.3 Metabolic pathway analysis and enrichment analysis

**Figure 7:**
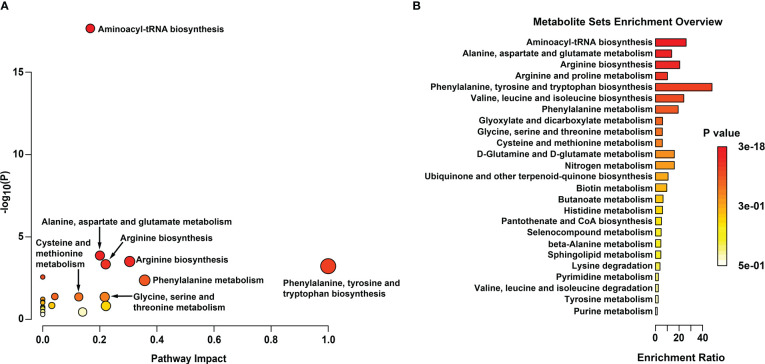
Metabolic pathway analysis **(A)** and enrichment analysis **(B)** of significant differential amino acids between HUVECs and HemECs by targeted metabolic analysis. In **(A)**, each related metabolic pathway is shown as a circle, in which the size and color are based on the pathway impact value and the P value, respectively.

All 16 significant differential amino acid metabolites were enriched to further investigate the metabolomic pathways involved in IH. As presented in **Figure 7A**, these significant DMs were enriched in 25 metabolic pathways (**Table S6**), among which eight metabolic pathways were significant with p < 0.05 and impact >0.1 in HemECs compared with HUVECs. These pathways included ‘aminoacyl-tRNA biosynthesis’, ‘alanine, aspartate and glutamate metabolism’, ‘arginine biosynthesis’, ‘arginine and proline metabolism’, ‘phenylalanine, tyrosine and tryptophan biosynthesis’, ‘valine, leucine and isoleucine biosynthesis’, ‘phenylalanine metabolism’, ‘glyoxylate and dicarboxylate metabolism’, ‘cysteine and methionine metabolism’, and ‘glycine, serine and threonine metabolism’ (**Table 4**). Within these pathways, ‘aminoacyl-tRNA biosynthesis’, ‘alanine, aspartate and glutamate metabolism’, and ‘arginine biosynthesis’ were highlighted as targets to investigate the pathological mechanism underlying HemEC metabolism. Metabolite set enrichment analysis showed that the top three pathways associated with HemEC metabolism were aminoacyl-tRNA biosynthesis; alanine, aspartate and glutamate metabolism; and arginine biosynthesis (**Figure 7B**).

**Table 4:** Eight significant pathways between HUVEC and HemEC by targeted metabolic analysis.

Pathway	Total	Expected	Hits	P value	Impact
Aminoacyl-tRNA biosynthesis	48	0.49548	13	2.23E-18	0.16667
Alanine, aspartate and glutamate metabolism	28	0.28903	4	0.00013	0.20032
Arginine biosynthesis	14	0.14452	3	0.00031	0.30457
Arginine and proline metabolism	38	0.39226	4	0.00045	0.22050
Phenylalanine, tyrosine and tryptophan biosynthesis	4	0.04129	2	0.00059	1
Phenylalanine metabolism	10	0.10323	2	0.00429	0.35714
Cysteine and methionine metabolism	33	0.34065	2	0.04379	0.12630
Glycine, serine and threonine metabolism	33	0.34065	2	0.04379	0.21707

## 4 Discussion

Several signaling pathways regulate angiogenesis during the development of IH, especially the VEGF/VEGF receptor (VEGFR) pathway and Notch pathway (7). During vessel sprouting, endothelial “tip” cell migration and endothelial “stalk” cell elongation are initiated under the control of VEGF and Notch signaling. Huge energy is then obtained to supply EC and maintain the process of sprouting angiogenesis. Several metabolic pathways, including glycolysis, fatty acid oxidation, and amino acid metabolism, participate in the regulation of angiogenesis (10). These metabolic pathways have different roles in the production of energy and biomass and the maintenance of redox homeostasis during EC angiogenesis (10). Recently, it was shown that inhibiting glucose metabolism in EC by targeting the key glycolytic enzyme PFKFB3 reduced pathological angiogenesis (19, 20). In addition, targeting glutamine metabolism inhibits EC migration during pathological angiogenesis (21), and fatty acid metabolism participates in regulating EC angiogenesis (22). Therefore, a better understanding of the metabolic changes in ECs will improve the beneficial effects of antiangiogenic therapy in IH.

Glucose is the most important metabolic substrate of EC metabolism. Targeting glucose metabolism by inhibiting glycolysis is a promising strategy to reverse EC dysfunction and inhibit pathological angiogenesis (23). Previous study has demonstrated that the genes expression of glycolytic enzymes including glucose transporter 1 (GLUT1), hexokinase 2 (HK2) and lactate dehydrogenase A (LDHA) are significant higher in human pulmonary artery endothelial cells (HPAEC) than in chronic thromboembolic pulmonary hypertension endothelial cells (CTEPH-EC) and pulmonary arterial hypertension endothelial cells (PAH-EC) (24). Recently, our previous study found that glucose metabolism pathways, including pyruvate metabolism and the citrate cycle, were significantly enriched between proliferating IH and involuting IH (14). In addition, inhibiting the key glycolytic enzyme phosphofructokinase 1 suppresses proliferation and migration and induces cell cycle arrest in HemEC (14). Furthermore, key glycolytic enzymes, including hexokinase 2,6-phosphofructo-2-kinase 3, pyruvate kinase M2 (PKM2) and lactate dehydrogenase A, are more highly expressed in HemECs than in HUVECs (25). PKM2 was demonstrated to play a critical role in regulating the progression of IH and the therapeutic resistance of propranolol treatment (26). Together, the results from these studies indicated that HemEC glucose metabolism participates in the development of IH (27). As mentioned previously, several metabolic pathways, including amino acid metabolism and fatty acid oxidation but not glycolysis, are involved in EC metabolism. However, no study has investigated the role of amino acid metabolism or fatty acid oxidation in IH until now. Therefore, a comprehensive understanding of the metabolic profiles of HemEC will improve our knowledge of the pathogenesis of IH.

The present study was performed with the aim of investigating the metabolic profiles of HemEC. Metabolomics is emerging as a powerful tool for studying metabolic processes, identifying crucial biomarkers responsible for metabolic characteristics and revealing metabolic mechanisms (28). In the present study, nontargeted metabolic analysis was first conducted to reveal the metabolic profiles of HemEC. The results showed a significant metabolic change between HUVECs and HemECs. In addition, these significant DMs were predominantly enriched in metabolic pathways related to amino acid metabolism, including ‘arginine and proline metabolism’ and ‘alanine, aspartate and glutamate metabolism’, consistent with a previous study (29). Therefore, it can be speculated that amino acid metabolism is involved in HemEC metabolism. Similar results are noted that amino acid pathways including D-glutamine/D-glutamate metabolism and arginine/proline metabolism were highly expressed in brain endothelial cell compared to primary astrocytes (30). In addition, amino acid pathways, such as arginine and proline metabolism, D-glutamine and D-glutamate metabolism and alanine, aspartate and glutamate metabolism, participated in the regulation of cardiovascular endothelial cells metabolism (31). Then, targeted metabolic analysis was further performed to compare the difference in amniotic acid between HUVECs and HemECs. The results of targeted metabolic analysis showed that most amino acid metabolites, including glutamine, asparagine, arginine and serine, were significantly upregulated in HemECs compared with HUVECs. These findings indicated that amino acid metabolism is much more active in HemEC. KEGG pathway enrichment indicated that these significant amino acids were ‘alanine, aspartate and glutamate metabolism’, ‘arginine and proline metabolism’ and ‘glycine, serine and threonine metabolism’. Recently, amino acid metabolism pathways, including ‘alanine, aspartate and glutamate metabolism’ and ‘arginine biosynthesis’, were significantly identified based on the differential metabolites in umbilical cord blood serum samples from IH (29). Our previous study found that ‘glycine, serine and threonine metabolism’ and ‘valine, leucine and isoleucine degradation’ were associated with the development of IH (14). In the present study, our targeted metabolic analysis results were consistent with those of previous studies (14, 29). In addition, amino acid metabolism pathways including ‘aminoacyl-tRNA biosynthesis’, ‘arginine and proline metabolism’, ‘alanine, aspartate and glutamate metabolism’ and ‘phenylalanine, tyrosine and tryptophan biosynthesis’ were identified simultaneously in both nontargeted metabolic analysis and targeted metabolic analysis. These findings indicated that these pathways play a more important role in HemEC metabolism. Taken together, these results suggest that amino acid metabolism participates in the regulation of HemEC metabolism.

Aminoacyl-tRNA synthetases can activate amino acids and cause them bind to tRNA and form the corresponding aminoyl-tRNA. Recently, emerging evidence has demonstrated that aminoacyl-tRNA synthetases participate in various functions, such as protein synthesis, gene regulation, intron splicing and angiogenesis (32). Thus, targeting aminoacyl-tRNA synthetases has been a promising treatment for infections, cancer and autoimmune diseases (33). Glutamine is the most abundant free amino acid in blood plasma. ECs from both venous and arterial vessels express glutaminase, a phosphate-dependent enzyme that converts glutamine into glutamate (14). When glutamine is converted into glutamate, glutamate is then used to produce a-ketoglutarate to refuel the TCA cycle. Glutamine deprivation or inhibition of glutaminase 1 can impair EC proliferation and migration and reduce pathological angiogenesis (34). In addition, glutamine is used to produce glutathione in ECs, which is used to maintain redox homeostasis. ECs are vulnerable to ROS-induced damage when glutamine is depleted (34, 35). In addition, glutamine can provide nitrogen for asparagine synthesis. When asparagine synthetase is silenced, EC proliferation is impaired because asparagine synthesis from glutamine-derived nitrogen and aspartate is inhibited (34).

Arginine, a semiessential amino acid, is an important substrate for endothelial nitric oxide synthase to generate the vasoprotective molecule nitric oxide (36). Nitric oxide can promote vessel growth through upregulating VEGF (37). Enhancing the basal production of nitric oxide by store-operated Ca^2+^ entry can accelerate HemEC proliferation, and propranolol treatment can decrease the level of nitric oxide to induce vasoconstriction (38, 39). In addition, arginine depletion inhibits angiogenesis in colon cancer, and dietary arginine supplementation suppresses the progression of colon cancer by increasing NO secretion (40). In the present study, the ‘valine, leucine and isoleucine biosynthesis’ pathway was identified as a significant metabolic pathway, indicating that branched-chain amino acids (BCAAs) are involved in HemEC metabolism. BCAAs are used for protein synthesis or are oxidized for energy purposes by tumors, and emerging evidence suggests that alterations in BCAA metabolism might be useful for therapy when considered in combination with genetic mutations^38^. These research results indicated that amino acid metabolism can regulate EC angiogenesis. However, the underlying mechanism of amino acid metabolism and its effect on the development of IH remains unknown. Therefore, additional studies are needed.

In conclusion, our results reveal that amino acid metabolism is involved in IH. Key differential amino acid metabolites, including glutamine, asparagine, arginine and serine, may play an important role in the regulation of angiogenesis in HemEC.

## Data availability statement

The original contributions presented in the study are included in the article/**Supplementary Material**. Further inquiries can be directed to the corresponding author.

## Ethics statement

The studies involving human participants were reviewed and approved by Ethics Committee of the West China Hospital of Sichuan University. The patients/participants provided their written informed consent to participate in this study.

## Author contributions

Conception and design: KY, TQ, YJ. Collection and assembly of data: KY, TQ, JZ, XG, YJ. Manuscript preparation: KY, TQ. Manuscript editing: KY, TQ. Manuscript revision/review: SC, YJ. Manuscript final version approval: All authors. All authors contributed to the article and approved the submitted version.
