# Supplementary material for: Integrated nontargeted and targeted metabolomics analyses amino acids metabolism in infantile hemangioma
Source: Front Oncol. 2023 Mar 21;13:1132344. doi: 10.3389/fonc.2023.1132344 (PMC10070834; doi:10.3389/fonc.2023.1132344)
Supplement: Supplementary file 1 [file DataSheet_1.zip › Supplementary Materials/Table S1 Clinical features of sin patients with proliferating infantile hemangioma.docx]

**Table S1. Clinical features of sin patients with proliferating infantile hemangioma**

| **Rank** | **Sex** | **Age** | **Location** | **Growth Phase** |
| --- | --- | --- | --- | --- |
| 1 | Female | 5 months | Back | Proliferation |
| 2 | Female | 7 months | Upper limb | Proliferation |
| 3 | Male | 5 months | Abdominal wall | Proliferation |
| 4 | Female | 4 months | Elbow | Proliferation |
| 5 | Male | 3 months | Neck | Proliferation |
| 6 | Female | 6 months | Thoracic wall | Proliferation |
