# Supplementary figures and images for: Integrated nontargeted and targeted metabolomics analyses amino acids metabolism in infantile hemangioma
Source: Front Oncol. 2023 Mar 21;13:1132344. doi: 10.3389/fonc.2023.1132344 (PMC10070834; doi:10.3389/fonc.2023.1132344)

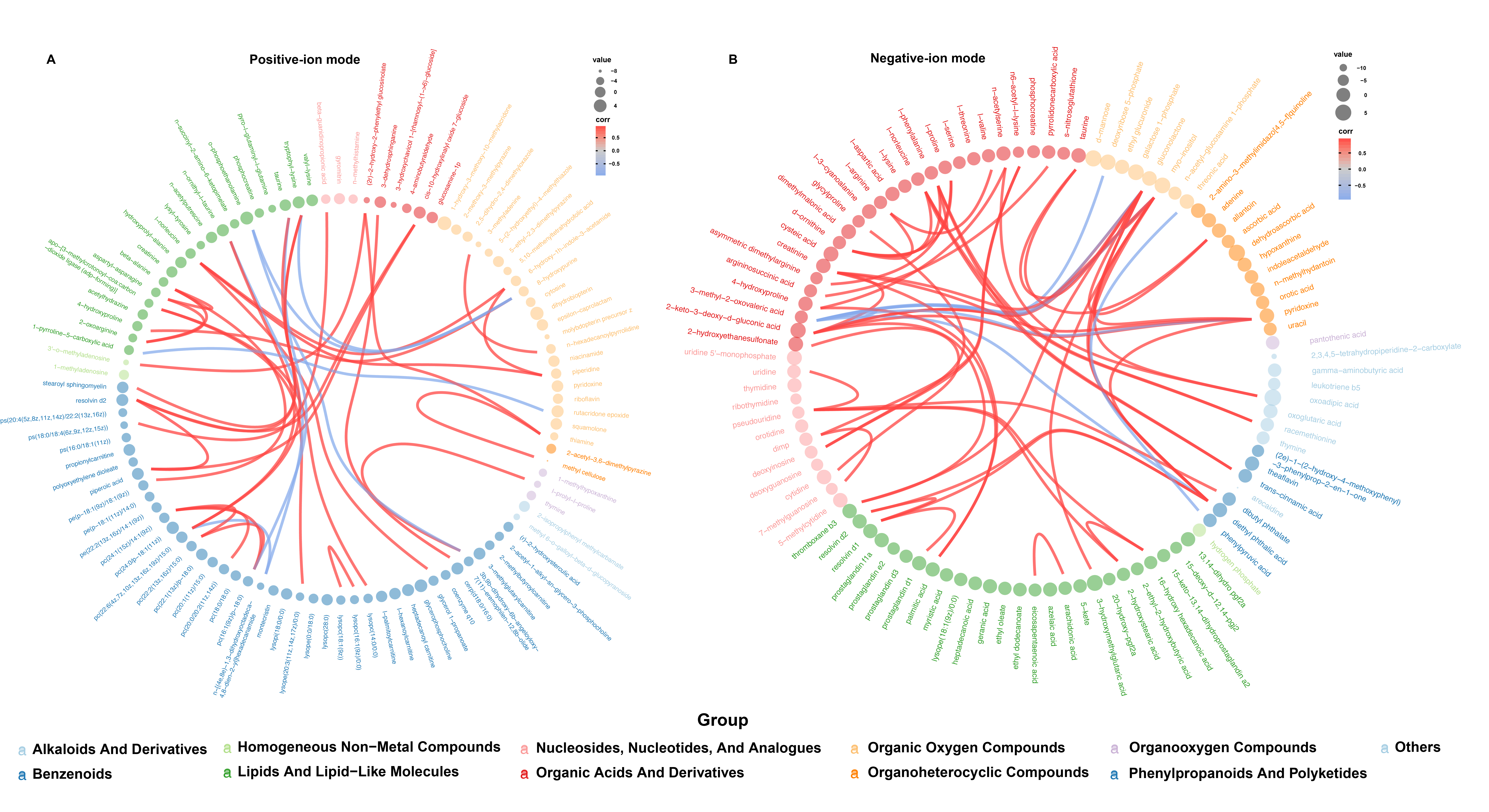

Supplement: Supplementary file 1 [file DataSheet_1.zip › Supplementary Materials/Figure S1 Chord diagrams showing the correlation between classifications and the content of differential metabolites.jpg]
